# Rapid Spread of Classical Swine Fever Virus among South Korean Wild Boars in Areas near the Border with North Korea

**DOI:** 10.3390/pathogens9040244

**Published:** 2020-03-25

**Authors:** SeEun Choe, Ra Mi Cha, Dae-Sung Yu, Ki-Sun Kim, Sok Song, Sung-Hyun Choi, Byung-Il Jung, Seong-In Lim, Bang-Hun Hyun, Bong-Kyun Park, Dong-Jun An

**Affiliations:** 1 Virus Disease Division, Animal and Plant Quarantine Agency, Gimchen, Gyeongbuk-do 39660, Korea; ivvi59@korea.kr (S.C.); rami.cha01@korea.kr (R.M.C.); kisunkim@korea.kr (K.-S.K.); ssoboro@naver.com (S.S.); saint78@korea.kr (S.-I.L.); hyunbh@korea.kr (B.-H.H.); parkx026@korea.kr (B.-K.P.); 2 Division of Veterinary Epidemiological, Animal and Plant Quarantine Agency, Gimchen, Gyeongbuk-do 39660, Korea; shanuar@korea.kr; 3 Korea Pork Producers Association, Seocho-gu, Seoul 06643, Korea; heechan9@hanmail.net (S.-H.C.); exksa001@daum.net (B.-I.J.); 4 College of Veterinary Medicine, Seoul University, Gwanak-ro, Gwanak-gu, Seoul 08826, Korea

**Keywords:** CSFV, wild boar, antibody, transmission, E2

## Abstract

There has been a rapid increase in the number of classical swine fever (CSF) sero-positive wild boars captured near the demilitarized zone (DMZ), located the border with North Korea. In 2015–2016, few CSFV-positive antibody boars were detected; however, the number has increased steeply since 2017. Most occurred in the northern region of Gyeonggi before spreading slowly to Gangwon (west to east) in 2018–2019. Multi-distance spatial cluster analysis provided an indirect estimate of the time taken for CSFV to spread among wild boars: 46.7, 2.6, and 2.49 days/km. The average CSF serum neutralization antibody titer was 4–10 (log _2_), and CSFV Ab B-ELISA PI values ranged from 65.5 to 111.5, regardless of the age and sex of wild boars. Full genome analysis revealed that 16 CSFV strains isolated from wild boars between 2017 and 2019 were identical to the YC16CS strain (sub-genotype 2.1d) isolated from an outbreak in breeding pigs near the border with North Korea in 2016. The rapid increase in CSF in wild boars may be due to a continuously circulating infection within hub area and increased population density. The distribution pattern of CSFV in Korean wild boars moves from west to southeast, affected by external factors, including small-scale hunting, geographical features and highways.

## 1. Introduction

Classical swine fever virus (CSFV) is a single-stranded RNA virus belonging to the genus pestivirus (family, Flaviviridae). Classical swine fever (CSF) is one of the most important viral diseases affecting domestic pigs and wild boars [1]. Wild boars are as susceptible to CSFV as domestic pigs; therefore, eradication of CSF from wild boars is of epidemiologic value because it can prevent spread among domestic animals [2]. In Germany, 59% of CSF cases in domestic pigs from 1993 to 1998 were transmitted by direct or indirect contact with wild boars [3]. Over the last decades, several European Union (EU) member states (including Germany, France, and Slovakia) were confronted with outbreaks among wild boar; these outbreaks had a clear tendency to establish endemicity [4,5]. Following EU legislation, surveillance was implemented to ensure that CSFV is not circulating and spreading within wild-boar populations. At the beginning of a CSF outbreak, the antibody prevalence within a population is far below 5%, and it can be several months until the threshold of 5% is reached [5,6]. A previous study suggests that a higher incidence of CSF among wild boars in a particular region is closely related to the population density [7]. A high density of wild boars, particularly young wild boars, drives CSF outbreaks [7]. Recently, CSF have been reported in Gifu Prefecture, affecting domestic pigs and wild boars since September 2019 in Japan [8,9]. The current circulating CSFV in Japan was identified moderate pathogenicity and most closely matched in nucleotide identity, with CSFVs recently isolated in China and Mongolia [8]. A space–time permutation analysis in Japan showed virus transmission spread (10.3 and 4.9 days/km) among wild boars in two significant clusters [9]. After overlaying of a map of habitat quality, approximately 82% and 75% of CSF notifications in two clusters were found in the areas with potential contact between pigs and wild boars [9]. Monitoring of CSF in 5620 Korean wild boars captured between 2010 and 2014 identified only seven animals with CSFV and 23 animals with CSFV antibodies [10]. CSFV (YC16CS strain) isolated from an outbreak in breeding pigs in the north of Gyeonggi in 2016 shows a high genetic similarity and the same sub-genotype (2.1d) as the CSFV (CW17WB) strain isolated from wild boars in 2017 [11]. The risk of CSFV transmission from wild boars to breeding pigs is clear [11]. Therefore, we attempted to identify the reasons underlying the rapid spread of CSF infection among wild boars to guide development of prevention measures.

## 2. Results

### 2.1. CSF Antibody Prevalence According to Province

Korean wild boars captured between 2016 and 2019 comprised 40.2% females, 48.2% males, and 11.6% unknown. Of these, 4.7% (132/2799) of females, 3.6% (123/3362) of males, and 2.3% (19/809) of unknown animals were positive for CSFV antibodies (Table 1). With respect to age, 34.2% (2387/6970) of captured boars were under 1 year old, and 39.7% (2771/6970) were 1–2 years old (Table 1). In addition, 2.1% (51/2387) of boars under 1 year old, 4.9% (136/2771) aged 1–2 years old, 6.1% (36/583) aged 2–3 years old, 8.6% (22/255) aged 3–4 years old, 4.2% (5/119) aged over 4 years, and 2.8% (24/855) of indeterminate age were positive for CSFV antibodies (Table 1). The CSF sero-positive rates in Gyeonggi (GG) increased continuously over the years: from 1.6% (5/302) in 2016 to 4.6% (6/129) in 2017, 9.2% (9/97) in 2018, and 14.3% (65/453) in 2019, as did the rates in Gangwon (GW) (from 0.6% (1/148) in 2016 to 4.7% (12/251) in 2017, 16.5% (33/200) in 2018, and 21.2% (129/608) in 2019) (Table 2). The number of CSF sero-positive wild boars of GW region in 2018 and 2019 was significantly different (*p* < 0.01 for 2018 and *p* < 0.001 for 2019), compared to that of other regions (Gyeongnam, Gyeongbuk, Jennam, Jenbuk, Chungnam, Chungbuk, Jeju, and Unknown), using two-way analysis of variance (ANOVA) with Bonferroni posttest (Table 2).

### 2.2. Genetic Analysis of CSFVs Isolated from Wild Boars

**Figure 1:**
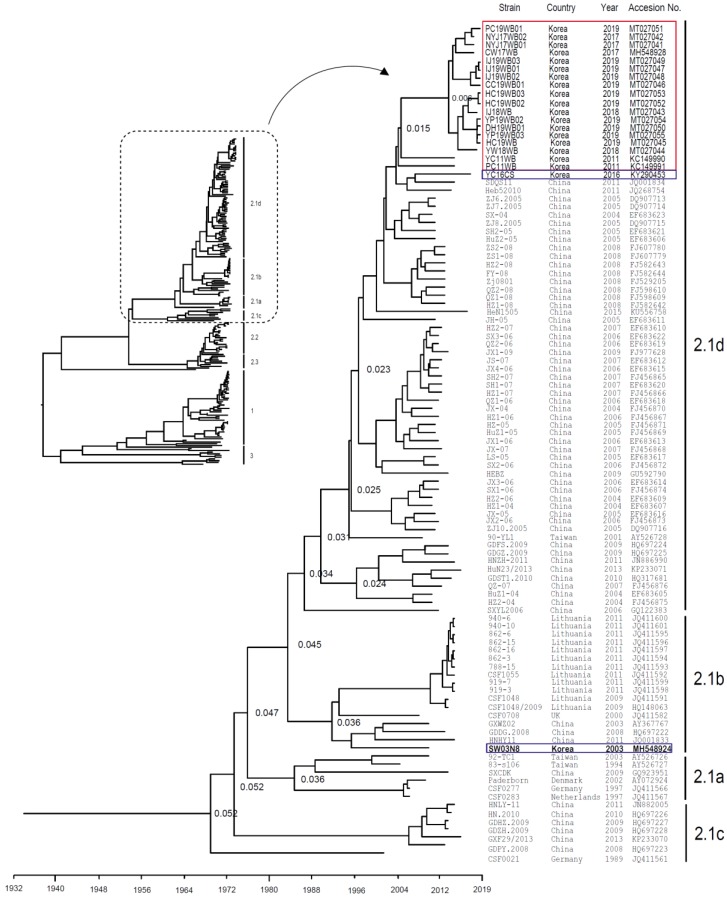
Phylogenetic analysis of the complete E2 gene sequence of 16 CSFV strains isolated from Korean wild boars (2017–2019). Complete E2 gene sequences (n = 197) were obtained from the NCBI GenBank database. Each dataset was simulated by using the following options: generation = 100,000,000; burn-in, 10%; and ESSs > 200. The confidence of the phylogenetic analysis-based timescale by factor (1.0) is represented by the numbers above the nodes representing branch length (time). Eighteen CSFV strains isolated from Korean wild boars (2011–2019) and two CSFV strains isolated from domestic pigs (2003 and 2016) are marked by red and blue boxes, respectively.

Between 2017 and 2019, we isolated 16 CSFV strains from Korean wild boars: three (NYJ17WB01, NYJ17WB02, and CW17WB) in 2017, two (YW18WB and IJ18WB) in 2018, and 11 (HC19WB, CC19WB01, IJ19WB01, IJ19WB02, IJ19WB03, DH19WB01, PC19WB01, HC19WB02, HC19WB03, YP19WB02, and YP19WB03) in 2019. Phylogenetic analysis revealed that all strains belonged to sub-genotype 2.1d (Figure 1). The mean time of the most recent common ancestor (tMRCA) for the Korean wild-boar CSFV strains was estimated to be 26.231 years ago (95% highest posterior density (HPD) interval, 22.1806–17.6393), with an effective sample size (ESS) of 276.3935 on the maximum clade credibility (MCC) tree. The clock rate (×10^−4^ substitutions/site/year) was 6.19, with a 95% HPD interval of 5.2898–7.2025 (Figure 1). Genetic Analysis of the complete genomes of the 16 Korean strains isolated in 2017–2019 revealed 99.2%–99.5% identity. Analysis of complete E2 sequences among the 16 strains revealed 98.4%–99.3% identity at the nucleotide (nt) level and 98.1%–99.5% identity at the amino acid (aa) level. The 16 strains isolated in 2017–2019 were 95.3%–96.3% (nt) and 97.3%–98.7% (aa), similar to the complete E2 gene of the YC11WB strain isolated from Korean wild boars in 2011; however, they were 98.6%–99.8% (nt) and 98.9%–100% (aa), similar to the YC16CS strain isolated in 2016 from Korean domestic pigs (Yeoncheon region).

### 2.3. Space–Time Clusters

**Figure 2:**
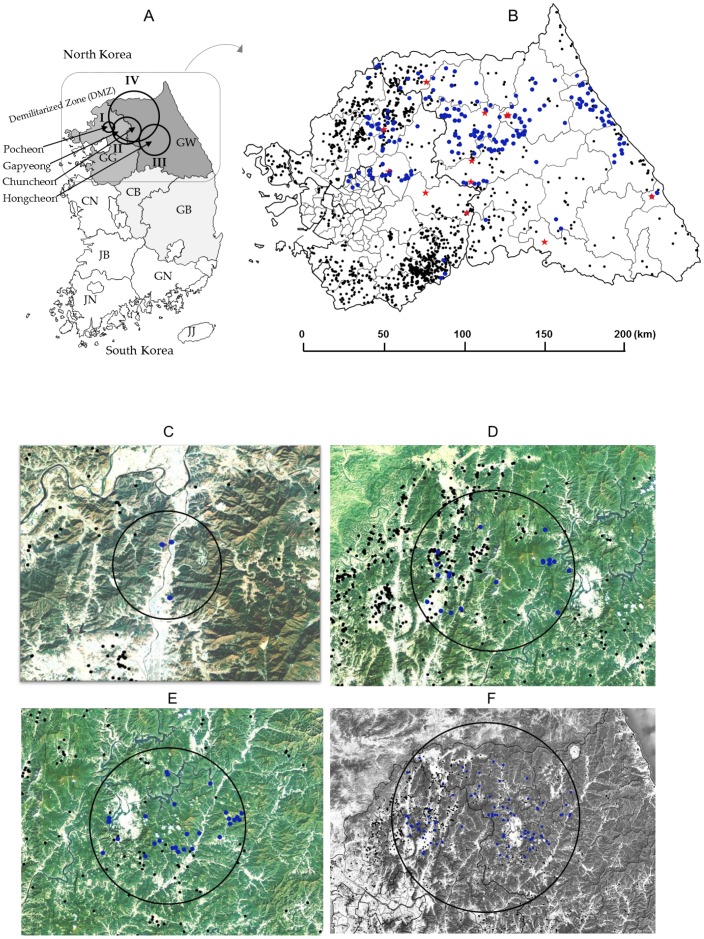
Space–time cluster analysis of CSF antibody-positive cases and antibody titers in wild boars. Positions of four clusters on the Korea map (**A**). Positions of pig farms (dark dot) and of CSF antibody (blue dot) and CSF antigen (red star) from captured wild boars between 2016 and 2019 (**B**). Space–time cluster analysis of CSF sero-positive wild boars was conducted, using SaTScan software (version 9.6), with the minimum time aggregation set as one month. Three clusters are marked with black circles (**C**: cluster I, **D**: cluster II, and **E**: cluster III). Space–time cluster analysis of CSF antibody titers was conducted by using data from the cluster IV marked with a black circle (**F**). Images (C,D, E,F) are marked dark dot (pig farm) and blue dot (CSF sero-positive wild boar). The nine regions (A) were as follows: GW: Gangwon; GG: Gyeonggi; GN: Gyeongnam; GB, Gyeongbu; JN: Jennam; JB: Jenbuk; CN: Chungnam; CB: Chungbuk; and JJ: Jeju.

**Figure 3:**
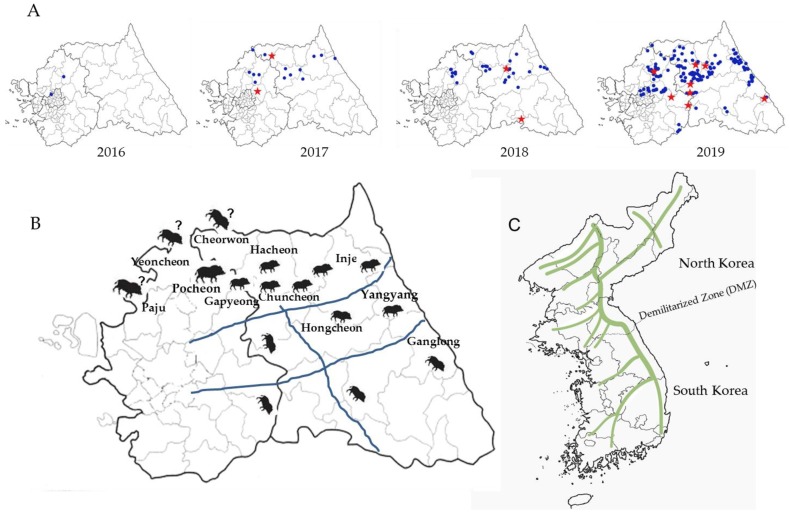
Predicted movements of CSFV-infected wild boars. Sites of capture of CSF antigen- and antibody-positive wild boars by year (2016–2019) are marked by a red star and blue circle, respectively (**A**). Expected routes and highways are marked by wild-boar pictures and blue lines (**B**). On the map of Korea, consecutive high mountains are marked by green lines (**C**).

Space–time permutation analysis identified three significant space–time clusters (*p* < 0.05) across South Korea (Table 3 and Figure 2A,C–E). Cluster I was in an area located in the northern part of country (Pocheon), with a radius of 3.87 km in which five CSF sero-positive wild boars were captured from 30th September 2017 to 29th March 2018 (Table 3 and Figure 2C). Two pig farms raise 1420 pigs in Cluster I. Cluster II, about 4.45 km southeast from Cluster I, the 22 CSF antibody-positive cases were detected within a 23.05 km (Gapyeong and Chuncheon) radius from 1st March 2019 to 29th April 2019 (Table 3 and Figure 2D). The 103 pig farms raise 119,346 pigs in Cluster II. Cluster III, which lies east of Cluster II, had a radius of 24.49 km (Hongcheon); 34 CSF sero-positive cases were captured here from 30th December 2018 to 28th February 2019 (Table 3 and Figure 2E). The 49 pig farms raise 54,690 pigs in Cluster III. Multi-distance spatial cluster analysis of the three Cluster regions estimated that the transmission time for CSFV among wild boars was about 46.7 days/km for cluster I, 2.6 days/km for Cluster II, and 2.49 days/km for Cluster III. Areas harboring boars with high CSFV antibody titers were also examined by using space–time permutation analysis (aggregate unit: one month). The data revealed one significant space–time cluster (*p* < 0.05); from 11th January 2017 to 31th October 2019, 107 cases were identified with high CSFV antibody titers within a 55.15 km (Cluster IV) radius (Figure 2A,F). The mean titer inside and outside log_2_ transformation of the Cluster IV were 6.94 and 7.99 log_2_, respectively, and standard deviation is 1.62 log_2_. The 395 pig farms raise 641,541 pigs in Cluster IV. Total of pig farms in Gangwon (n = 264) and Gyeonggi (n = 1249) is 1513 (Figure 2B). The number of pigs is approximately 485,875 in Gangwon and 1,913,234 in Gyeonggi, respectively. CSF antigen- and antibody-positive wild boars by year (2016–2019) were gradually expanded from west to east (Figure 3A). CSFV-infected wild boars are predicted to move along the mountain range (Figure 3B,C).

### 2.4. Relationship among CSF Sero-Positive and Age

**Figure 4:**
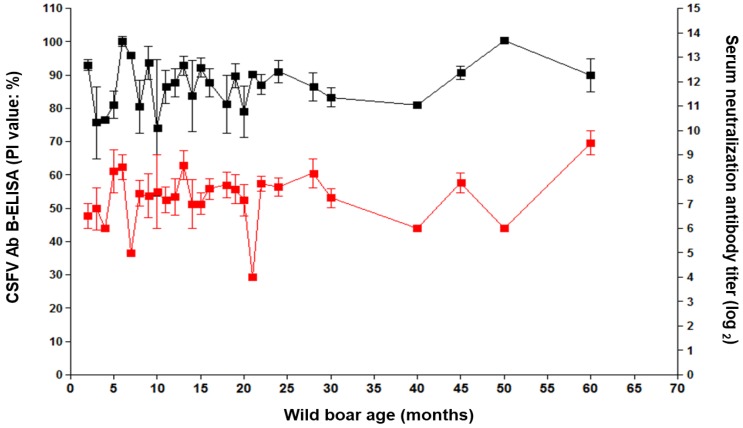
Relationship between CSF sero-positive and wild-boar age. CSFV antibodies were measured by PI value (≥ 40% positive and < 40% negative), using the CSFV Ab B-ELISA (black line) and titers (log_2_), using the serum neutralization antibody test (red line). Data are expressed as the mean ± SD (standard deviation).

From January 2016 to December 2019, 274 wild boars were confirmed as CSF sero-positive (Figure 4). Age and antibody titer (calculated from the serum neutralization antibody test and CSFV AB B-ELISA results) of CSFV-positive wild boars showed a close relationship (Figure 4). PI values for all antibody-positive wild boars ranged from 65.5 to 111.5 in the antibody ELISA and from 4 to 10 log_2_ in the serum neutralization antibody test (Figure 4). In boars aged < 5 months, the SN titer ranged from 6 to 7 log_2_, and that in wild boars aged 60 months ranged to 9.5 log_2_ (Figure 4).

## 3. Discussion

To investigate the increase of CSF among wild boars in Korea, we mainly used CSF antibody detection and consider that CSF sero-positive wild boars as CSFV affected animal. Two CSF sero-positive wild boars were identified in wild boars from the Pocheon region, in the second half of 2016; since then, the number of positive cases has increased rapidly, spreading from east to west, between 2017 and 2019. The temporal clustering of CSF infection in wild boars is consistent with animal movements during the mating and breeding seasons, mainly late autumn and early spring (i.e., November and March); this period involved increased contact between susceptible animals and infected hosts. In addition, the radius of spatial–temporal clusters is heavily influenced by habitat fragmentation caused by roads, railways, and pipelines [12]. The average CSF transmission time per km in Cluster I (Pocheon) was 46.7 days, compared with 2.6 days for Cluster II (Gapyeong and Chuncheon) and 2.49 days for Cluster III (Hongcheon). The differences are thought to be due to differences in wild-boar population density. The population density of wild boars in Pocheon increased sharply from 0.8 in 2016 to 3.9 in 2017, after which the density in neighboring Gapyeong to the east increased from 1.1 in 2017 to 5.3 in 2018, and that in Chuncheon increased from 3.3 in 2017 to 6.7 in 2018 [13]. As the number of wild boars within a certain habitat increased, CSF spread could be predicted indirectly based on animal movements. Paju, Yeoncheon, and Cheorwon are adjacent to the demilitarized zone (DMZ), meaning that North Korean wildlife can cross into these areas over the mountain range. Pocheon, which lies just below these three regions, is a good wild-boar habitat and can act as a reservoir of CSFV. The first CSF antigens were detected in wild boars from Yeoncheon and Pocheon in 2011; since then, antibody-positive cases have been detected continuously within the area [10]. Interestingly, the CSFV strain (YC16CS) isolated from a breeding pig farm in Yeoncheon in 2016 is genetically identical to the 16 CSFV strains isolated from wild boars in 2017–2019 [11]. This suggested that CSFV could be infected mutually between wild boars and breeding pigs [11]. The risk of spreading CSF from wild boars to breeding pigs is much higher west region because more pig farms were present inside Cluster II (west), rather than Cluster III, in the east region in this study. Generally, pig farms are more concentrated in Northern Gyeonggi Province (Paju, Yeoncheon, Pocheon, etc.) than in Gangwon province. Nevertheless, pig farms are also distributed in Hongcheon (Cluster III) and Chuncheon (east of Cluster II), and the risk of spread from wild boars to breeding pigs was present. In Japan, directional distribution of CSF notifications from September 2018 to June 2019 showed movement toward the northeast direction [9]. The 16 CSFVs from Korean wild boars spread from west to east between 2017 and 2019, and CSF sero-positive wild boars also showed similar pattern (west to east). Interestingly, of the 18 CSFV strains belonging to sub-genotype 2.1d detected since 2011, the two strains (YC11WB and PC11WB) identified in 2011 were slightly different from the 16 strains isolated between 2017 and 2019 [11]. This may be due to genetic mutations caused by self-circulating infection among Korean wild boars over six years; however, it could be due to the crossing of other strains from North Korea into the DMZ. A previous study suggested that the persistence of CSFV infection among wild boars is due to a combination of virus characteristics (e.g., pathogenicity) and the size of the wild-boar population [2]. It was known that, a small population (less than 2000) of wild boars would promote self-limit on the spread of CSFV within one year, whereas CSFV tends to persist and become endemic for years, in larger populations [7].

In September 2019, in South Korea, African swine fever virus (ASFV) was endemic in breeding pigs; the cause of the disease was believed to be wild boars [14]. For CSFV, South Korean experts also confirmed that the virus had spread from North Korean wild boars that had crossed the DMZ. To control transmission of ASF from wild boars, the wire fences running west to east were installed close to the DMZ, to prevent wild boars from crossing over. Between these two sets of wire fences, large-scale hunting was carried out, using firearms. As a result, 306 cases of ASFV were identified, with cases moving along the DMZ from west to east [14]. Because the Korean government began a large-scale hunting policy at the end of 2019, to control wild boars and reduce the risk of ASF, we expect that the number of CSFV antigen- and antibody-positive cases in 2020 will be less than that in 2019; this will tell us whether mass wild-boar hunting is an effective control measure for CSFV. The biggest obstacle to large-scale hunting in Korea is the inability to hunt in national parks. National parks have the possibility to act as uncontrolled disease reservoirs; therefore, controlling disease with different strategies, such as the installation of a protective fence in national parks, should be considered.

The effectiveness of using the hunting policy to control the disease spread was still controversial. Several reports suggested that hunting wild boars has reduced CSF occurrence [6,15]. In 1997, CSF was detected in wild boars in Northern Italy; therefore, the governments of Italy and Switzerland began a joint program to capture wild boars, leading to a marked reduction in the incidence CSF [6]. CSF sero-positive rates in these regions fell sharply, from 42.2% to 8.8%, suggesting no CSFV transmission between wild boars [6]. However, a recent study suggested that intensifying hunting or erecting fences has not been adequate for preventing disease spread or persistence [16] and also suggested that oral mass vaccine (OMV) has proved to be effective in maintaining herd immunity and achieving CSF control and it is the only available method for CSF eradication in large forested areas [16]. The European Food Safety Authority (EFSA) suggests that the reduction of the wild-boar population (targeted hunting of female wild boar) and carcass removal to stop the spread of ASFV in the wild-boar population are more effective when applied preventively in the infected area [17]. In order to decrease the spread of CSF among wild boars in Korea, we already try to reduce the wild-boar population by large-scale hunting policy and can install a fence to delay the transmission of CSF. In addition, CSF bait vaccine to maintain herd immunity will be sprayed in Gangwon and Gyenggi province from 2020. The CSF bait vaccine with DIVA function was developed to base the Flc-LOM-BE^rns^ vaccine strain [18]. Baiting of wild boars with the Flc-LOM-BE^rns^ vaccine will induce production of anti-CSF E2 antibodies and anti-BVDV E^rns^ antibodies simultaneously, but no anti-CSF E^rns^ antibodies [18], which make differential diagnosis of vaccinated animal from wild-type CSFV-infected animal.

After CSFV spread from west to east, we expected it to descend south along the mountains. We did not observe southward spread in 2018, although it did spread gradually in 2019. We suspect that the main reason for this spread pattern is physical obstacles, e.g., major roads crossing from west to east. The information obtained from wild-boar CSFV-surveillance studies will help to predict the path of spread in wild boars and protect domestic pig farms from CSFV. Moreover, studying the pattern of CSF occurrence in wild boars will be a valuable guide to predicting the route and direction of ASFV spread to the south from the nearby DMZ.

In conclusion, a rapid increase of CSF sero-positive in Korean wild boars may result from the circulating infections and increased population density within the hub area. In addition, the distribution of the CSFV in wild boars gradually spread from the west to the southeast and was affected by obstacles, such as small-scale hunting, geographical features, highways, and wire fences.

## 4. Materials and Methods

### 4.1. Sample Collection, RT-PCR, and Phylogenetic Analysis

From 2010, wild boars were hunted in co-operation with the Korean Pork Producers Association and the Korean government, to satisfy the OIE requirements for surveillance of wild boars and feral pigs in CSF-free countries. Blood samples were collected from 6970 wild boars hunted in nine provinces (Gangwon, Gyeonggi, Gyeongnam, Gyeongbuk, Jennam, Jenbuk, Chungnam, Chungbuk, and Jeju), in South Korea, between February 2016 and November 2019 (Table 1). Blood was collected in heparinized tubes. Total RNA was extracted by using a micro-column-based RNeasy Mini kit (Qiagen, CA, USA). The RT-PCR conditions and specific primers used to amplify the complete E2 gene have been reported previously [11,19]. Complete E2 gene sequences for CSFVs were obtained from the NCBI GenBank database and aligned by using the CLUSTAL X alignment program. A BEAST input file was then generated, using BEAUti within the BEAST package v1.8.1 [20]. Rates of nucleotide substitution per site and per year, and the tMRCA, were estimated by using a Bayesian MCMC approach. The exponential clock and expansion growth population model in the BEAST program was used to obtain the best-fit evolutionary model, and the MCC tree was visualized by using Figtree 1.4 [21].

### 4.2. Spatiotemporal Cluster Analysis

Space–time permutation scan statistics [22] were calculated retrospectively, to identify the presence of space–time clusters for geographical localities in which CSFV antibodies were detected in wild boars (*Sus scrofa*); there were 274 sites from 1st January 2016 to 31th December 2019. Space–time cluster analysis was conducted by using SaTScan software version 9.6 (Kulldor, Boston, MA, USA), with the minimum time aggregation set at 1 month (accounting for the minimum duration of CSFV antibody persistence), a maximum spatial cluster size set as 50% of the population at risk, and a maximum spatial cluster size set as 50% of the total study period [9]. Test statistics were calculated for 999 Monte Carlo replications, to identify candidate clusters with statistical significance (*p* = 0.05).

### 4.3. CSFV Ab B-ELISA and SN Tests

Serum samples from wild boars were tested in a CSFV E2 Antibody ELISA. The CSFV Ab B-ELISA (BioNote Co. Cat. No. EB4413PO, Korea) is a competition ELISA designed to detect the E2 protein; however, it also provides a PI value (≥40% positive and <40% negative). Serum neutralization (SN) tests based on a neutralizing peroxidase-linked assay (NPLA) were performed to detect CSFV-specific neutralizing antibodies. Briefly, PK-15 cells inoculated with CSFV were incubated for 72 h, at 37 °C/5% CO_2_, with serum samples (serially diluted 2-fold). PK-15 cells were fixed in prechilled 80% acetone and then reacted with a 3B6 monoclonal antibody specific for CSFV E2 3B6 (Median Diagnostics Co., South Korea). PK-15 cells were then stained, using a VECTOR kit (Vector Laboratories, Burlingame, CA, USA), biotinylated anti-mouse IgG (H+L) (Cat. No. BA-9200), ABC solution (Cat. No. PK-4000), and a DAB peroxidase substrate (Cat. No. SK-4100). Staining was observed under a microscope.

### 4.4. Statistical Analysis

All statistical analyses were performed by using GraphPad Prism software, version 6.0, for Windows. Data were analyzed by using two-way analysis of variance (ANOVA) with Bonferroni posttest.

## Figures and Tables

**Table 1:** CSF sero-positive, gender, and age of wild boars captured from 2016 to 2019.

Year	No. of AP^a^ /No. of CWB^b^	Gender			Age (Months)					
		Male	Female	UK^c^	0–12	13–24	25–36	37–48	48–70	UK^c^
2016	7/1683	2/584	1/417	4/682	0/369	3/499	0/88	0/26	0/19	4/682
2017	20/1670	9/912	11/757	0/1	3/630	16/762	0/171	0/70	1/36	0/1
2018	47/1320	28/740	19/580	0/0	14/479	23/608	4/138	6/64	0/31	0/0
2019	200/2297	84/1126	101/1045	15/126	34/909	94/902	32/186	16/95	4/33	20/172
Total	3.9 ^d^ (274/ 6970)	3.6 (123/ 3362)	4.7 (132/ 2799)	2.3 (19/ 809)	2.1 (51/ 2387)	4.9 (136/ 2771)	6.1 (36/ 583)	8.6 (22/ 255)	4.2 (5/ 119)	2.8 (24/ 855)

Numbers in parenthesis denote CSF antibody-positive animals. AP^a^: antibody positive. CWB^b^: captured wild boar. UK^c^: unknown. ^d^Positive percentage (%) (No. of antibody positive/No. of wild boars tested).

**Table 2:** CSF sero-positive and region in which wild boars were captured from 2016 to 2019.

Year	No. of AP^a^ /No. of CWB^b^	Percentage (%) for Region (No. of Antibody Positive/No. of Wild Boars Tested)									
		GW	GG	GN	GB	JN	JB	CN	CB	JJ	UK^c^
2016	7/1683	0.6 (1/148)	1.6 (5/302)	0 (0/334)	0 (0/410)	0 (0/71)	0 (0/59)	0.3 (1/261)	0 (0/96)	0 (0/2)	0 (0/0)
2017	20/1670	4.7 (12/251)	4.6 (6/129)	0.5 (1/195)	0.3 (1/301)	0 (0/189)	0 (0/112)	0 (0/221)	0 (0/270)	0 (0/0)	0 (0/2)
2018	47/1320	16.5^d^ (33/200)	9.2 (9/97)	0.5 (1/196)	0.8 (2/237)	0 (0/150)	0 (0/97)	0 (0/172)	1.3 (2/149)	0 (0/22)	0 (0/0)
2019	200/2297	21.2^e^ (129/608)	14.3^f^ (65/453)	0.3 (1/292)	0.3 (1/275)	1.4 (1/69)	0 (0/71)	0.3 (1/289)	0.9 (2/203)	0 (0/37)	0 (0/0)

GW: Gangwon; GG: Gyeonggi; GN: Gyeongnam; GB, Gyeongbuk; JN: Jennam; JB: Jenbuk; CN: Chungnam; CB: Chungbuk; JJ: Jeju. Numbers in parentheses denote CSF antibody-positive animals. AP^a^: antibody positive. CWB^b^: captured wild boar. UK^c^: unknown. ^d^*p* < 0.01 and ^e^*p* < 0.001: CSF sero-positive of GW region in 2018 and 2019 was compared with other regions (GN, GB, JN, JB, CN, CB, JJ, and UK) in 2018 and 2019. ^f^*p* < 0.05: CSF sero-positive of GG region in 2019 was compared with other regions (GN, GB, JN, JB, CN, CB, JJ, and UK) in 2019.

**Table 3:** Spatiotemporal cluster analysis of CSF antibody distribution in captured Korean wild boars. Aggregate unit: one month.

		Spatiotemporal Cluster Analysis of CSF Antibody Distribution	
Cluster	I	II	III
Observed notifications	5	22	34
Expected notifications	0.35	6.84	11.59
Duration (days)	181	60	61
Start date	30/11/2017	01/03/2019	30/12/2018
End date	29/05/2018	29/04/2019	28/02/2019
Radius (km)	3.87	23.05	24.49
*p*-value	0.038	0.0018	<0.001
